# Treatment specific competence predicts outcome in cognitive therapy for social anxiety disorder

**DOI:** 10.1016/j.brat.2012.09.001

**Published:** 2012-12

**Authors:** Denise M. Ginzburg, Christiane Bohn, Volkmar Höfling, Florian Weck, David M. Clark, Ulrich Stangier

**Affiliations:** a Department of Clinical Psychology and Psychotherapy, University of Frankfurt, Frankfurt, Germany; b Department of Experimental Psychology, University of Oxford, Oxford, UK

**Keywords:** Treatment competence, Treatment integrity, Social anxiety disorder, Cognitive therapy

## Abstract

Several studies have demonstrated a positive relationship between competence and outcome in CBT for depression but studies of CBT for anxiety disorders are lacking. The present study explores the relationship between competence and outcome in cognitive therapy (CT) for social anxiety disorder, using hierarchical linear modeling analyses (HLM). Data were drawn from a multicenter randomized controlled trial. Five trained raters evaluated videotapes of two therapy sessions per patient using the Cognitive Therapy Competence Scale for Social Phobia (CTCS-SP). Overall adherence to the treatment manual and patient difficulty were also assessed. Patient outcome was rated by other assessors using the Clinical Global Impression Improvement Scale (CGI-I) and the Liebowitz Social Anxiety Scale (LSAS). Results indicated that competence significantly predicted patient outcome on the CGI-I (*β* = .79) and LSAS (*β* = .59). Patient difficulty and adherence did not further improve prediction. The findings support the view that competence influences outcome and should be a focus of training programs. Further research is needed to compare different ways of assessing competence and to understand the complex relationships between competence and other therapy factors that are likely to influence outcome.

## Highlights

► We studied for the first time the effect of therapy competence on outcome in CBT for social anxiety disorder. ► A treatment specific therapy competence scale showed high inter-rater reliability. ► Competence predicted outcome, even when controlling for adherence and patient difficulty. ► Achieving high levels of competence should be a target of therapist training courses.

## Introduction

The strong evidence base for cognitive behavior therapies (see Nathan & Gorman, 2007; Roth & Fonagy, 2004) has led to their inclusion in treatment guidelines issued by professional groups (such as American Psychological Association Division 12: Chambless & Ollendick, 2001; German Psychological Association: Heinrichs, Stangier, Gerlach, Willutzki, & Fydrich, 2010) and national bodies (such as the UK National Institute for Clinical Excellence: www.nice.org). Building on these guidelines, several countries are developing large-scale dissemination programs that aim to greatly increase the public availability of CBT and many other countries are considering doing so (Berge, 2011; Clark, 2011). If these dissemination programs are to be a success, it will be necessary for researchers to specify the key CBT skills that need to be taught and to develop measures to assess whether the skills have been acquired. Roth and Pilling (2008) have published the key competencies required for a range of effective CBTs for depression and various anxiety disorders. Less progress has been made in developing measures to determine whether a trainee has successfully acquired these competencies.

The most common way of assessing competence is by rating videotapes of therapy sessions. Webb, Derubeis, and Barber (2010) recently reported a meta-analytic review of 17 studies that related competence to outcome. A significant relationship was observed for the treatment of depression, but not other clinical conditions. In addition, no studies of CBT for anxiety disorders were identified. A likely reason for the more positive results in depression is that the most commonly used measure was the Cognitive Therapy Scale (CTS: Young & Beck, 1980), which was originally developed for the NIMH Collaborative Depression trial (Elkin et al., 1989).

The present study focuses, for the first time, on the relationship between competence and outcome in a CBT for anxiety. The treatment chosen for investigation is cognitive therapy (CT) for social anxiety disorder, based on Clark and Wells' (1995) model. Randomized controlled trials have established that CT is an effective intervention that compares favorably to exposure therapy (Clark, Ehlers, et al., 2006), interpersonal psychotherapy (Stangier, Schramm, Heidenreich, Berger, & Clark, 2011) two types of group CBT (Mörtberg, Clark, Sundin, & Aberg, 2007; Stangier, Heidenreich, Peitz, Lauterbach, & Clark, 2003), SSRIs (Clark et al., 2003) and medication-based treatment as usual (Mörtberg et al., 2007). Experimental studies (McManus et al., 2009) have provided further support for the view that part of CT's effectiveness is likely to be due to procedures that are treatment specific. The competence measure was the Cognitive Therapy Competence Scale for Social Phobia (CTCS-SP; Clark, Von Consbruch, Hinrichs, & Stangier, 2006), which is a modified version of the CTS in which many original items were reworded and new items were introduced in order to assess the specific competencies required in this treatment approach. As patient difficulty and adherence to a treatment protocol may also determine outcome, each of these were also assessed. The data is drawn from the CT arm of Stangier et al.'s (2011) trial.

## Method

### The clinical trial

The trial was conducted at the Outpatient Clinics of the Department of Psychology, University of Frankfurt, Germany and the Department of Psychiatry, University of Freiburg, Germany. Patients who met DSM-IV criteria for social anxiety disorder were randomized to CT, interpersonal psychotherapy or no-treatment (Wait). For a detailed description of the trial, including the comparative outcome findings, see Stangier et al. (2011). The current study focuses on data from the CT-patients. Patients received up to 16 weekly sessions over four months.

### Treatment

CT was based on Clark and Wells' (1995) model of the maintenance of SAD and followed a detailed German manual (Stangier, Clark, & Ehlers, 2006). Treatment was essentially the same as that used in previous trials on CT (Clark et al., 2003; Clark, Ehlers, et al., 2006; Mörtberg et al., 2007; Stangier et al., 2003). The treatment comprised the following interventions: 1) establishing a personal model using patient's beliefs, images, safety behaviors, focus of attention, and symptoms; 2) role-play based behavioral experiments to demonstrate the adverse effects of self-focused attention and safety behaviors; 3) practicing externally focused attention in social and nonsocial situations; 4) restructuring distorted self-imagery using video feedback and other methods; 5) conducting surveys to gather information to challenge dysfunctional beliefs; and 6) behavioral experiments to test negative beliefs in anxiety-provoking social situations while dropping safety behaviors and recreating feared outcomes (e.g sweating, blushing, displaying ignorance, etc).

### Participants

A total of 38 patients (20 male) were assigned to CT. Mean age was 34.8 years (SD = 9.16; range = 20–62 years). Thirty-one patients completed treatment and seven (18.4%) dropped out Videotapes of the therapy sessions were available from all completers and three drop-outs.

### Therapists

CT was delivered by 10 therapists (2 male, 8 female) with an average of 4.8 (SD = 2.9) years of clinical experience (range: 2–12 years), each of whom had either completed their psychotherapy training or were at an advanced stage in the training. Their age ranged from 29 to 51 years (*M* = 33.7, SD = 7.0) and they had an average of 1.5 years (SD = 2.3) experience in the treatment of social anxiety disorder. Prior to the trial, the therapists received 30 h of training in CT for social anxiety disorder and treated at least two pilot cases. All therapists received regular supervision from experienced supervisors throughout the trial. The average number of patients treated by each therapist was 3.4 (SD = 1.9, range 1–6).

### Measures

Following the recommendations of Webb et al. (2010) and Perepletchikova (2009), clinical outcome was rated by assessors who were blind to whether the patient had received treatment and competence was rated by different assessors who were blind to clinical outcome.

#### Clinical outcome

The primary outcome measure is treatment response on the Clinical Global Impression Improvement Scale (Guy, 1976) as modified for use in social anxiety disorder (Liebowitz et al., 1992; Stein et al., 1998). The CGI-I is a seven point rating scale of improvement from baseline, ranging from 1 (“very much improved”) to 7 (“very much worse”). This scale assesses overall psychological functioning, symptoms of SAD and disability and has been shown to have good reliability and validity (Zaider, Heimberg, Fresco, Schneier, & Liebowitz, 2003). The CGI-I was completed at the end of treatment by trained and experienced raters. A secondary outcome measure was the Liebowitz Social Anxiety Scale (LSAS; Liebowitz, 1987; Stangier & Heidenreich, 2005), which was completed by the same raters before and after treatment. The LSAS consists of 24 items on two separate scales, assessing fear (ranging from 0 = none to 3 = severe) and avoidance (ranging from 0 = never to 3 = usually). Good psychometric properties of the LSAS have been repeatedly demonstrated (Cronbach's alpha = .96; Fresco et al., 2001; Heimberg et al., 1998). Inter-rater reliability for the LSAS in the present trial, based on interviews with 17 patients, was excellent (*r* = .97, *p* < .001). Assessors completed the LSAS before the CGI-I.

#### Competence

The Cognitive Therapy Competence Scale for Social Phobia (CTCS-SP: Clark, Ehlers, et al., 2006) comprises 16 items (see Table 1) which assess how well specific components of treatment were implemented. The components are rated on a seven point scale ranging from 0 to 6. A mean score is computed for the 16 specific items. Von Consbruch, Clark, and Stangier (2012) reported satisfactory psychometric properties for the CTCS-SP. Inter-rater reliabilities for the mean competence score on the scale were good (ICC = .81). The internal consistency of the scale was high (*α* = .97) and the test-retest reliability excellent (*r*_tt_ = .92 for the mean CTCS-SP score and *r*_tt_ = .55–.96 for the single items).

#### Adherence and patient difficulty

Adherence refers to the extent to which the techniques that the therapist uses are those recommended in the therapy manual (Stangier et al., 2006). It does not require a judgment of how well they are implemented. CT adherence was judged by a single item that ranged from 0 (not adherent) to 6 (very adherent). Patient difficulty was also assessed by a single item that ranged from 0 (not difficult) to 6 (very difficult). Von Consbruch et al. (2012) reported good inter-rater reliability for both items: ICC = .79 for adherence and .75 for patient difficulty.

#### Raters

CTCS-SP, adherence and patient difficulty were rated by five PhD candidates and clinical psychologists, who had an average of 3.4 years experience with CT and had treated an average of six patients each (SD = 1.4). All raters received at least two days of training and had to evaluate a minimum of 15 practice tapes and to achieve consensus before starting to rate the study videotapes. In order to minimize rater drift, all five raters met regularly throughout the study to discuss sample tapes and review discrepancies.

#### Materials

Videotapes were available from 34 out of 38 patients. Two tapes were selected for each patient with one being randomly selected from the middle of therapy (sessions 4–13), and one being selected from either the beginning or the end (sessions 1–3 & 14–16). Sixty-eight treatment sessions (12.5% of the total sessions) were each independently evaluated by two raters. The mean of the four ratings per patient (two from the first session and two from the second session) were used in the analyses (*N* = 34 mean ratings per patient). Therapists did not know which sessions would be selected for rating. If a tape from a particular session was missing, an adjacent session was used.

#### Data analysis

Intraclass correlation coefficients (ICCs) model 1 (ICC_(1,*n*)_; see Shrout & Fleiss, 1979) were computed to analyze the inter-rater reliabilities for all items in the competence (CTCS-SP) scale as well as the adherence item and patient difficulty. The ICCs were calculated on the mean of two judgments (ICC_(1,2)_) on the basis of all videotapes (*N* = 68). The 95% confidence interval was used to determine statistical significance. According to Portney and Watkins (2009) ICCs ≥ .75 can be categorized as good, those between .75 and .50 are considered moderate and <.50 is not satisfactory.

Mean competence ratings for the therapy that each patient received were computed by averaging the ratings for the two videotaped sessions. Mean adherence and patient difficulty ratings were computed in the same way.

To determine whether competence, adherence and/or patient difficulty predicted clinical outcome, hierarchical linear modeling (HLM, Bryk & Raudenbush, 1992) with random intercept was carried out with M*plus* version 6 (Muthén & Muthén, 2010) applying the maximum likelihood (ML) estimator. Separate HLM models were computed for two outcome variables (CGI-I and LSAS). In the model for LSAS, residualized gain scores (LSASres) were used to take into account differences in pre-treatment scores. All three potential predictors (competence, adherence and patient difficulty) were entered into each model. As patients were nested within therapists, two 2-level models were specified with patients at level 1 and therapists at level 2. The following fit statistics were used: Root Mean Square Error of Approximation (RMSEA) and Standardized Root Mean Square Residual (SRMR) to estimate overall fit, and comparative fit Index (CFI) to estimate the incremental fit. Within *Mplus* terminology (Muthén & Muthén, 2010), *β* represents the effects of a predictor variable on a criterion (outcome) variable.

## Results

### Inter-rater reliability of the competence, adherence and patient difficulty scales

Inter-rater reliabilities were computed using the full set of tapes (*N* = 68). The ICC for the mean competence score (items 1–16) was .84 (*p* < .001). For individual competence items ICCs ranged from .60 to .92. The ICCs for adherence and patient difficulty were .83 (*p* < .001) and .67 (*p* < .001) respectively.

### Means for competence, adherence, patient difficulty and clinical outcome

The mean competence level for the therapy sessions that each patient received was moderate (*M* = 2.9, SD = .52, on a scale that ranges from 0 to 6), as was the average level of adherence (*M* = 3.7, SD = .85, on a scale that ranges from 0 to 6). Mean patient difficulty was low (*M* = .95, SD = .74, on a scale ranging from 0 to 6). Patient improvement on CGI was moderate to high (*M* = 2.0, SD = 1.14 where 1 is “very much improved” and 7 is “very much worse”). Mean LSAS was 68.5 (SD = 24.3) at pre-treatment and 37.0 (SD = 21.2) at post-treatment. The mean change in LSAS was 30.5 (SD = 19.3) and the pre-treatment to post-treatment effect size was 1.26.

### Prediction of clinical outcome by competence, adherence and patient difficulty

**Fig. 1:**
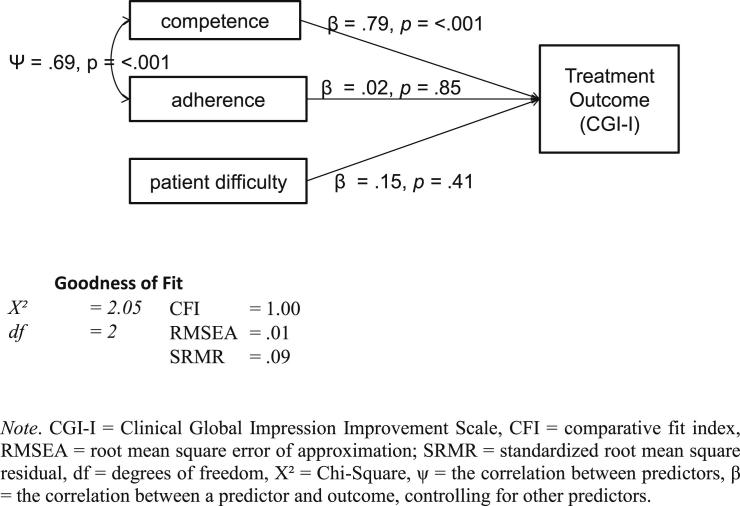
Path analysis model with random intercept, competence (CTCS-SP), adherence and patient difficulty as predictors and treatment outcome (CGI-I) as the dependent variable.

The HLM models relating predictors to outcome produced acceptable fit statistics with both the CGI-I (*χ*^2^ = 2.05, df = 2, *p* = .32; CFI = 1.00, RMSEA = .01, SRMR = .09) and LSASres (*χ*^2^ = 2.07, df = 3, *p* = .56; CFI = 1.00, RMSEA = .00, SRMR = .097). The HLM model accounted for 48% of the variance in the primary outcome variable (CGI-I). Only competence was a significant predictor of CGI-I (*β* = .79, *p* = <.001). *β* values relating adherence and patient difficulty to CGI-I were .02 (*p* = .85) and .15 (*p* = .41) respectively. The HLM model for the secondary outcome measure (LSASres) explained 20% of the outcome variance. Again, competence was the only significant predictor (*β* = .59, *p* = .01). The *β* values for adherence and patient difficulty were .09 (*p* = .59) and .24 (*p* = .20) respectively. Including an indirect path between competence and outcome via patient difficulty reduced the model goodness of fit, suggesting that the strong link between competence and outcome was not due to patient difficulty influencing both (Fig. 1).

### Exploratory item analysis of the CTCS-SP

A further analysis was conducted to determine whether a subset of items on the competence scale were particularly important as predictors of outcome. Correlations were computed between the 16 individual competence items and the CGI, with significance levels adjusted for multiple comparisons (Bonferroni correction). Table 1 shows the results.

Of the 6 individual competence items that significantly predict outcome, four are specific to the CT for social anxiety disorder competence scale (e.g. “focus on social-phobia-related cognitions, self-focused attention and imagery”; “selection of appropriate strategies for change in social-phobia-related cognition and maintaining factors”; “appropriate implementation of techniques for change in social-phobia-related cognition and maintaining factors” and “resource activation”), and two (“interpersonal effectiveness” & “pacing and efficient use of time”) are more general CT items whose phrasing was modified to take into account the typical presentation of patients with social anxiety disorder and the emphasis of the treatment on behavioral experiments.

## Discussion

Several studies have demonstrated significant, but modest, correlations between competence and outcome in CBT for depression (Webb et al., 2010). As far as we are aware, this is the first study to demonstrate such a relationship within a CBT for an anxiety disorder. A strong relationship was observed between competence as assessed by the CTCS-SP and outcome in CT for social anxiety disorder, with 48% of the variance in the primary outcome measure (CGI-I) being explained.

Our study has several methodological strengths. Outcome and competence were assessed by different raters, with the assessors of outcome also being blind to whether the patient had received treatment. Two sessions per patient, taken from different phases of treatment, were assessed for competence, a manual for rating competence was developed, raters completed a formal training programme based on the manual and practiced rating a substantial number of tapes before the study commenced. This training is likely to have contributed to the good inter-rater reliabilities that were obtained with the CTCS-SP.

The CTCS-SP is a modified version of Young and Beck's (1980) cognitive therapy scale (CTS). Many items were re-written to take into account the challenges of treating patients with social phobia (e.g., social withdrawal and use of safety behaviors in sessions) and the specialized procedures that are used (videofeedback, attention training, distinctive behavioral experiments, etc). It may be that the CTCS-SP was successful at predicting outcome because of it's specific focus on CT for social anxiety disorder. The item-by-item analysis would appear to be consistent with this view, as is the evidence (see Introduction) that at least part of the effectiveness of CT is likely to be attributable to procedures that are a distinctive feature of the treatment. However, a formal comparison between the CTCS-SP and the more general CTS (or CTS-R) is required in order to assess the relative predictive power of specific versus more general measures of CBT competence.

Although ratings of actual therapy sessions are the most common method for assessing competence, some training courses and research groups use standardized role-plays. Future research could usefully focus on their comparative utility. One might argue that tape ratings are a more direct assessment of what a therapist actually does with a client. However, role-plays have the potential advantage that situations are standardized, so the trainer can ensure key features of therapy are always sampled and patient characteristics are controlled. With ratings of actual sessions it is always possible that some therapists may appear more competent because they are treating more compliant patients, who are in turn generally more likely to have a better outcome. The fact that ratings of patient difficulty did not predict outcome suggests this was not a major complication in the present study. However, we accept that there may have been some other patient characteristic that we did not measure that partly mediated the relationship between competence ratings and outcome.

Competence presupposes a reasonably high level of adherence in the sense that one cannot be judged to have implemented a treatment well if the procedures specified in the manual were not used. Given this point, one might expect a relationship between adherence and outcome. However, in our HLM analyses adherence was not a significant predictor. As is common in randomized controlled trials, adherence was good and showed little variability (see Waller, 2009). This may partly explain the negative finding. In routine clinical settings adherence is likely to be more variable and hence may play a larger role in predicting outcome. In the present study, patient difficulty was not itself a significant predictor of outcome, perhaps because variability was low. However, this will not always be the case (Foley, O'Malley, Rounsaville, Prusoff, & Weissman, 1987).

The association between competence and outcome was somewhat higher for the primary outcome measure CGI-I (*β* = .79), than for the LSASres (*β* = .59). The CGI covers both the formal symptoms of social anxiety disorder and the impact that the disorder has on the individual's life in general, whereas the LSAS only assesses situational fear and avoidance. The broader focus of the CGI-I is probably closer to the focus of therapy and it could be argued that it is more appropriate as a dependent variable when assessing whether competence predicts patient outcomes.

An important practical implication of our findings is that it would be helpful to use competence ratings as part of the assessment of a therapist's progress and benefit from CBT training programs for social anxiety disorder. In order to disseminate evidence-based psychological treatments (Clark et al., 2009; McHugh & Barlow, 2010; Shafran et al., 2009), psychotherapists need to be trained to deliver treatments competently. Our findings would seem to support the importance of training therapists to deliver the treatment specific procedures in a competent manner.

The rigorous requirements of a research investigation into the relationship between competence and outcome do not necessarily apply to the use of competency ratings to facilitate routine training programs or clinical practice. For example, although it was useful for our study to have each tape independently rated by two assessors, this is unlikely to be needed for training courses once a reasonable level of agreement between assessors in the team has been established. The assessment of competence on the basis of specific segments rather than the whole videotaped session (Weck, Bohn, Ginzburg, & Stangier, 2011) could also be explored as an alternative to address time and cost issues. Finally, informal use of the cognitive therapy rating scale by students themselves is likely to be helpful. Certainly, we have found that many therapists who are learning CT for social anxiety learn a great deal about how particular procedures should be implemented by studying the particular items on the CTCS-SP and rating their own sessions according to the scale.

### Limitations

The study has several limitations. First, we did not control for a number of other therapy factors that might also have influenced outcome, such as empathy, warmth and positive regard of the therapist (Keijsers, Schaap, & Hoogduin, 2000), therapeutic alliance and patient motivation (Huppert, Barlow, Gorman, Shear, & Woods, 2006). These confounds are an inherent limitation of observational data and should, ideally, be either controlled for, or investigated as mediators/moderators in future studies (Castonguay, Goldfried, Wiser, Raue, & Hayes, 1996; Feeley, DeRubeis, & Gelfand, 1999). Second, our sample size of 34 patients treated by 10 therapists is modest. Replication with a larger overall sample and more patients per therapist would be desirable.

## Conflict of interest

None reported.

## Funding

This research was supported by grants STA 512/2-1/2 from the German Research Foundation (Deutsche Forschungsgemeinschaft) (Dr Stangier) and 069777 from the Wellcome Trust and by the National Institute for Health Research Biomedical Research Centre at the South London and Maudsley Health Service Foundation Trust and Kings College London (Dr Clark).

## Figures and Tables

**Table 1:** Competence components and their relation to outcome on CGI-I.

Items on CTCS-SP	CGI-I
1. Agenda setting	−.35
2. Dealing with questions/objections/problems	−.43
3. Clarity of communication	−.46
4. Pacing and efficient use of time	−.65**
5. Interpersonal effectiveness	−.51**
6. Resource activation	−.61**
7. Reviewing social-phobia questionnaires and other measures	−.21
8. Reviewing previously set homework	−.31
9. Use of feedback and summaries	−.22
10. Guided discovery	−.48
11. Focus on social-phobia-related cognitions, self-focused attention, safety behaviors, and biased imagery	−.49*
12. Rationale	−.36
13. Selection of appropriate strategies for change in social-phobia-related cognition and maintaining factors (including selection of behavioral experiments and other experiential exercises).	−.59**
14. Appropriate implementation of techniques for change in social-phobia-related cognition and maintaining factors (including selection of behavioral experiments and other experimental exercises).	−.56**
15. Integration of discussion and experiential techniques	−.34
16. Setting homework	−.23

*Note*. CGI-I = Clinical Global Impression Improvement Scale; CTCS-SP = Cognitive Therapy Competence Scale for Social Phobia. *p < .05; **p < .01. Significance levels were adjusted for multiple comparisons (Bonferroni correction).

## References

[bib1] Berge, T. (2011). *Improving access to psychological therapies in Europe*. Symposium presented at 41st annual congress of the European Association of Behavioral and Cognitive Therapies, Reykjavik, Iceland, 01.09.2011.

[bib2] Bryk A.S., Raudenbush S.W. (1992). Hierarchical linear models: Applications and data analysis methods.

[bib3] Castonguay L.G., Goldfried M.R., Wiser S., Raue P.J., Hayes A.M. (1996). Predicting the effect of cognitive therapy for depression: a study of unique and common factors. Journal of Consulting and Clinical Psychology.

[bib4] Chambless D.L., Ollendick T.H. (2001). Empirically supported psychological interventions: controversies and evidence. Annual Review of Psychology.

[bib5] Clark D.M. (2011). Implementing NICE guidelines for the psychological treatment of depression and anxiety disorders: the IAPT experience. International Review of Psychiatry.

[bib6] Clark D.M., Ehlers A., Hackmann A., McManus F., Fennell M., Grey N. (2006). Cognitive therapy versus exposure and applied relaxation in social phobia: a randomized controlled trial. Journal of Consulting and Clinical Psychology.

[bib7] Clark D.M., Ehlers A., McManus F., Hackmann A., Fennell M., Campbell H. (2003). Cognitive therapy versus fluoxetine in generalized social phobia: a randomized placebo-controlled trial. Journal of Consulting and Clinical Psychology.

[bib8] Clark D.M., Layard R., Smithies R., Richards D.A., Suckling R., Wright B. (2009). Improving access to psychological therapy: initial evaluation of two UK demonstration sites. Behaviour Research and Therapy.

[bib9] Clark, D. M., Von Consbruch, K., Hinrichs, S., & Stangier, U. (2006). Cognitive therapy competence scale for social phobia (CTCS-SP), unpublished manuscript. 10.1017/S1352465811000622 22047669

[bib10] Clark D.M., Wells A., Heimberg R., Liebowitz M.R., Hope D.A., Schneier F.R. (1995). A cognitive model of social phobia. Social phobia: Diagnosis, assessment and treatment.

[bib11] Elkin I., Shea M.T., Watkins J.T., Imber S.D., Sotsky S.M., Collins J.F. (1989). National Institute of Mental Health treatment of depression collaborative research program. General effectiveness of treatments. Archives of General Psychiatry.

[bib12] Feeley M., DeRubeis R.J., Gelfand L.A. (1999). The temporal relation of adherence and alliance to symptom change in cognitive therapy for depression. Journal of Consulting and Clinical Psychology.

[bib13] Foley S.H., O'Malley S., Rounsaville B., Prusoff B.A., Weissman M.M. (1987). The relationship of patient difficulty to therapist performance in interpersonal psychotherapy of depression. Journal of Affective Disorders.

[bib14] Fresco D.M., Coles M.E., Heimberg R.G., Liebowitz M.R., Hami S., Stein M.B. (2001). The Liebowitz social anxiety scale: a comparison of the psychometric properties of self-report and clinician-administered formats. Psychological Medicine.

[bib15] Guy W. (1976). ECDEU assessment manual for psychopharmacology.

[bib16] Heimberg R.G., Liebowitz M.R., Hope D.A., Schneier F.R., Holt C.S., Welkowitz L.A. (1998). Cognitive behavioral group therapy vs phenelzine therapy for social phobia: 12-week outcome. Archives of General Psychiatry.

[bib17] Heinrichs N., Stangier U., Gerlach A., Willutzki U., Fydrich T. (2010). Evidenzbasierte Leitlinie zur Psychotherapie der Sozialen Angststörung.

[bib18] Huppert J.D., Barlow D.H., Gorman J.M., Shear M.K., Woods S.W. (2006). The interaction of motivation and therapist adherence predicts outcome in cognitive behavioral therapy for panic disorder: preliminary findings. Cognitive and Behavioral Practice.

[bib19] Keijsers G.P., Schaap C.P., Hoogduin C.A. (2000). The impact of interpersonal patient and therapist behavior on outcome in cognitive-behavior therapy. A review of empirical studies. Behavior Modification.

[bib20] Liebowitz M.R. (1987). Social phobia. Modern Problems in Pharmacopsychiatry.

[bib21] Liebowitz M.R., Schneier F., Campeas R., Hollander E., Hatterer J., Fyer A. (1992). Phenelzine vs atenolol in social phobia. A placebo-controlled comparison. Archives of General Psychiatry.

[bib22] McHugh R.K., Barlow D.H. (2010). The dissemination and implementation of evidence-based psychological treatments. American Psychologist.

[bib23] McManus F., Clark D.M., Grey N., Wild J., Hirsch C., Fennell M. (2009). A demonstration of the efficacy of two of the components of cognitive therapy for social phobia. Journal of Anxiety Disorders.

[bib24] Mörtberg E., Clark D.M., Sundin O., Aberg W.A. (2007). Intensive group cognitive treatment and individual cognitive therapy vs. treatment as usual in social phobia: a randomized controlled trial. Acta Psychiatrica Scandinavica.

[bib25] Muthén L.K., Muthén B.O. (2010). Mplus user's guide.

[bib26] Nathan P.E., Gorman J.M. (2007). A guide to treatments that work.

[bib27] Perepletchikova F. (2009). Treatment integrity and differential treatment effects. Clinical Psychology-Science and Practice.

[bib28] Portney L.G., Watkins M.P. (2009). Foundations of clinical research: Applications to practice.

[bib29] Roth A.D., Fonagy P. (2004). What works for whom? A critical review of the psychotherapy research.

[bib30] Roth A.D., Pilling S. (2008). Using an evidence-based methodology to identify the competences required to deliver effective cognitive and behavioural therapy for depression and anxiety disorders. Behavioural and Cognitive Psychotherapy.

[bib31] Shafran R., Clark D.M., Fairburn C.G., Arntz A., Barlow D.H., Ehlers A. (2009). Mind the gap: improving the dissemination of CBT. Behaviour Research and Therapy.

[bib32] Shrout P.E., Fleiss J.L. (1979). Intraclass correlations – uses in assessing rater reliability. Psychological Bulletin.

[bib33] Stangier U., Clark D.M., Ehlers A. (2006). Soziale Phobie. Fortschritte der Psychotherapie Band 28.

[bib34] Stangier U., Heidenreich T., Collegium Internationale Psychiatriae Scalarum (2005). Liebowitz Soziale Angst Skala. Internationale Skalen für Psychiatrie.

[bib35] Stangier U., Heidenreich T., Peitz M., Lauterbach W., Clark D.M. (2003). Cognitive therapy for social phobia: individual versus group treatment. Behaviour Research and Therapy.

[bib36] Stangier U., Schramm E., Heidenreich T., Berger M., Clark D.M. (2011). Cognitive therapy vs interpersonal psychotherapy in social anxiety disorder a randomized controlled trial. Archives of General Psychiatry.

[bib37] Stein M.B., Liebowitz M.R., Lydiard R.B., Pitts C.D., Bushnell W., Gergel I. (1998). Paroxetine treatment of generalized social phobia (social anxiety disorder): a randomized controlled trial. JAMA.

[bib38] Von Consbruch K., Clark D.M., Stangier U. (2012). Assessing therapeutic competence in cognitive therapy for social phobia: psychometric properties of the cognitive therapy competence scale for social phobia (CTCS-SP). Behavioural and Cognitive Psychotherapy.

[bib39] Waller G. (2009). Evidence-based treatment and therapist drift. Behaviour Research and Therapy.

[bib40] Webb C.A., Derubeis R.J., Barber J.P. (2010). Therapist adherence/competence and treatment outcome: a meta-analytic review. Journal of Consulting & Clinical Psychology.

[bib41] Weck F., Bohn C., Ginzburg D.M., Stangier U. (2011). Assessment of adherence and competence in cognitive therapy: comparing session segments with entire sessions. Psychotherapy Research.

[bib42] Young J., Beck A.T. (1980). The development of the cognitive therapy scale.

[bib43] Zaider T.I., Heimberg R.G., Fresco D.M., Schneier F.R., Liebowitz M.R. (2003). Evaluation of the clinical global impression scale among individuals with social anxiety disorder. Psychological Medicine.

